# Classification of EEG Signals Based on Pattern Recognition Approach

**DOI:** 10.3389/fncom.2017.00103

**Published:** 2017-11-21

**Authors:** Hafeez Ullah Amin, Wajid Mumtaz, Ahmad Rauf Subhani, Mohamad Naufal Mohamad Saad, Aamir Saeed Malik

**Affiliations:** Centre for Intelligent Signal and Imaging Research (CISIR), Department of Electrical and Electronic Engineering, Universiti Teknologi Petronas, Seri Iskandar, Malaysia

**Keywords:** feature extraction, feature selection, machine learning classifiers, electroencephalogram (EEG)

## Abstract

Feature extraction is an important step in the process of electroencephalogram (EEG) signal classification. The authors propose a “pattern recognition” approach that discriminates EEG signals recorded during different cognitive conditions. Wavelet based feature extraction such as, multi-resolution decompositions into detailed and approximate coefficients as well as relative wavelet energy were computed. Extracted relative wavelet energy features were normalized to zero mean and unit variance and then optimized using Fisher's discriminant ratio (FDR) and principal component analysis (PCA). A high density EEG dataset validated the proposed method (128-channels) by identifying two classifications: (1) EEG signals recorded during complex cognitive tasks using Raven's Advance Progressive Metric (RAPM) test; (2) EEG signals recorded during a baseline task (eyes open). Classifiers such as, K-nearest neighbors (KNN), Support Vector Machine (SVM), Multi-layer Perceptron (MLP), and Naïve Bayes (NB) were then employed. Outcomes yielded 99.11% accuracy via SVM classifier for coefficient approximations (A5) of low frequencies ranging from 0 to 3.90 Hz. Accuracy rates for detailed coefficients were 98.57 and 98.39% for SVM and KNN, respectively; and for detailed coefficients (D5) deriving from the sub-band range (3.90–7.81 Hz). Accuracy rates for MLP and NB classifiers were comparable at 97.11–89.63% and 91.60–81.07% for A5 and D5 coefficients, respectively. In addition, the proposed approach was also applied on public dataset for classification of two cognitive tasks and achieved comparable classification results, i.e., 93.33% accuracy with KNN. The proposed scheme yielded significantly higher classification performances using machine learning classifiers compared to extant quantitative feature extraction. These results suggest the proposed feature extraction method reliably classifies EEG signals recorded during cognitive tasks with a higher degree of accuracy.

## Introduction

Clinicians use the electroencephalogram (EEG) as a standard neuroimaging tool for the study of neuronal dynamics within the human brain. Data extracted from EEG results reflect the process of an individual's information processing (Grabner et al., 2006). Recent technological advances have increased the scope of EEG recording abilities by using dense groups of electrodes including arrays of 128, 256, and 512 electrodes attached to the cranium. Visual inspection of these massive data sets is cumbersome (Übeyli, 2009) for existing EEG-based analysis techniques. Hence, optimized feature extraction of relevant EEG data is essential to improve the quality of cognitive performance evaluations, especially since it directly impacts a classifier's performance (Iscan et al., 2011). In addition, more expressive features enhance classification performance; hence, feature extraction has become the most critically significant step in EEG data classification.

Several researchers investigated “Quantitative-EEG” (QEEG) for the evaluation of neural activity during cognitive tasks. They used time and frequency domain features such as, entropy, power spectrum, autoregressive coefficients, and individual frequency bands, etc. (Doppelmayr et al., 2005; Zarjam et al., 2012). Frequency characteristics depend on neuronal activity and are grouped into several bands (delta, theta, alpha, beta, and gamma; Amin and Malik, 2013) that have been linked to cognitive processes.

Several feature extraction methods have been reported in the literature. These include time-frequency domain and the wavelet transform (WT) (Iscan et al., 2011). WT-based analysis is highly effective for non-stationary EEG signals compared to the short-time Fourier transformation (STFT). Moreover, wavelet-based features, including wavelet entropy (Rosso et al., 2001), wavelet coefficients (Orhan et al., 2011), and wavelet statistical features (mean, median, and standard deviations) have been reported for the evaluation of normal EEG patterns and for clinical applications (Yazdani et al., 2009; Garry et al., 2013). However, significant gaps in the literature exist regarding cognitive load studies and approaches to pattern recognition. Many studies employed multiple cognitive tasks such as, multiplication, mental object rotation, mental letter composing, and visual counting (Xue et al., 2003). These tasks are simple in nature and may not induce a high enough load to activate correlative cognitive neuronal networks that generate detectable electrical potentials. Furthermore, from a pattern recognition perspective, several studies excluded “feature normalization” and “feature selection” steps (Xue et al., 2003; Daud and Yunus, 2004), while many others used very few instances (observations) as input for classifiers that query the output of classification algorithms (Lin and Hsieh, 2009; Guo et al., 2011). In addition, some studies failed to cross validate their proposed classification procedures.

The present study utilized “Raven's Advance Progressive Metric” (RAPM)—commonly used to measure inter-individual differences in cognitive performance—as its standard psychometric cognitive task to stimulate EEG data (Raven, 2000; Neubauer and Fink, 2009). RAPM requires higher cognitive processing resources and reasoning ability to perform its tasks, which are non-verbal psychometric tests that require inductive reasoning considered an indicator of cognitive performance (Raven, 2000). The authors propose a “pattern recognition” approach comprising feature extraction, feature normalization, feature selection, feature classification, and cross validation (**Figure 5**). Wavelet coefficients were extracted using the discrete wavelet transform (DWT) as well as relative sub-band energies, which were then standardized to zero mean and unit variance. The feature selection process utilized statistical methods including Fisher's discriminant ratio (FDR) and principal component analysis (PCA) to eliminate non-significant features, which, in turn, optimized the “features” data set. As for classification, we employed K-nearest neighbors (KNN), Support Vector Machine (SVM), Multi-layer Perceptron (MLP), and Naïve Bayes (NB) to optimize the “features” set even further for our purpose of EEG pattern classification. Results were then compared with existing quantitative methods to confirm rather robust outcomes. Two EEG datasets were used to validate the proposed method. Dataset I comprise complex cognitive task (class 1) and baseline eyes open (class 2) task; while dataset II comprises two cognitive tasks: mental multiplication (class 1) and mental letter composing (class 2).

Section Materials and Methods describes materials and methods, followed by section Experimental Results and Discussion, which presents results and discussion, followed by section Limitations and concluding remarks.

## Materials and methods

This section provides details of experimental tasks and the dataset used in this study. In addition, we briefly describe the classification algorithms employed and the DWT, as well as the computations used for wavelet and relative energy.

### Raven's advance progressive matric test (RAPM)

**Figure 1:**
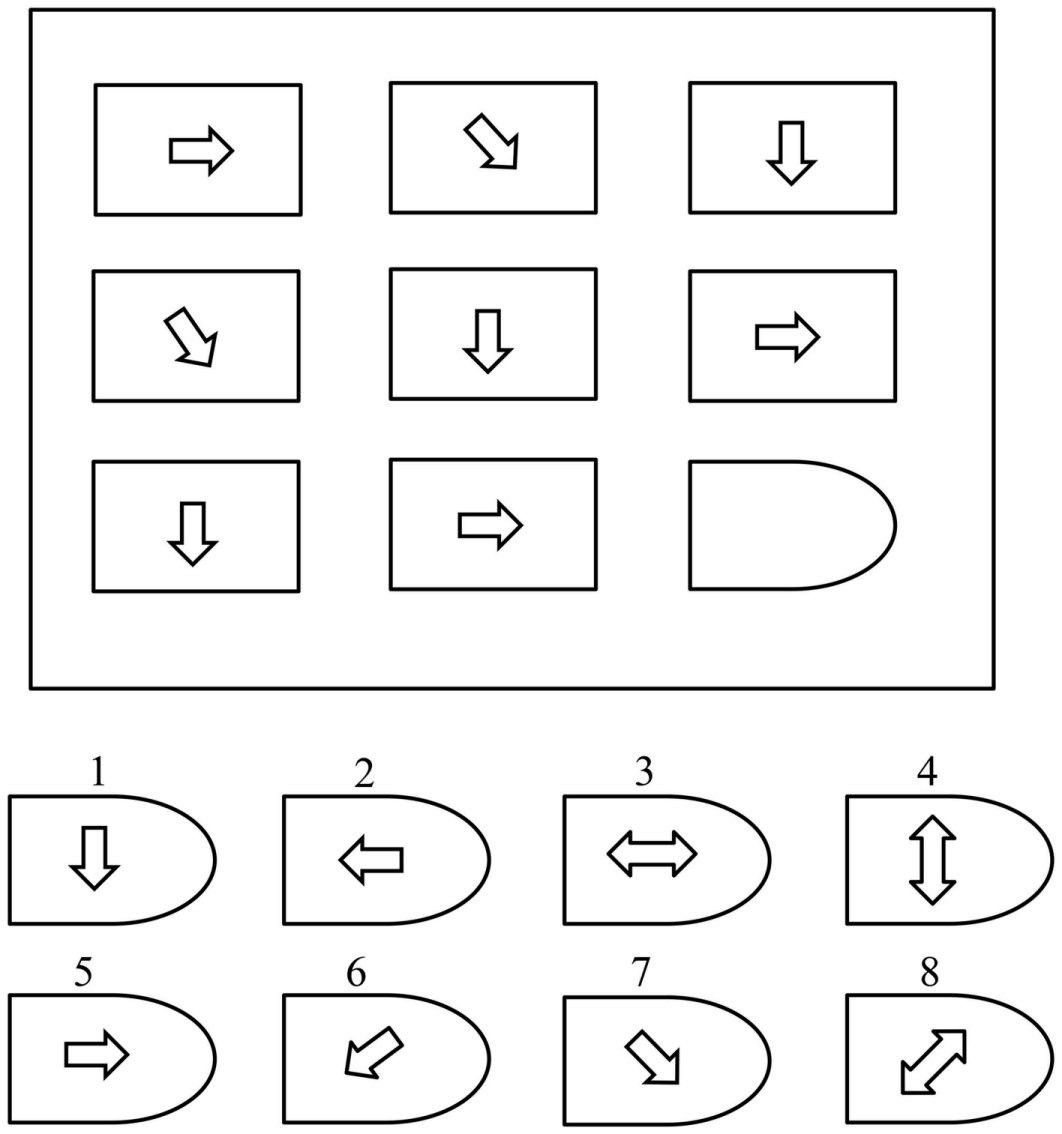
A simple Raven's style pattern (Amin et al., 2015b).

Raven's Advance Progressive Matric Test (Raven, 2000) is a non-verbal tool that measures levels of an individual's intellectual ability. It is commonly used to explicitly measure two components of general cognitive ability (Raven, 2000): “*the ability to draw meaning out of confusion, and the ability to recall and reproduce information that has been made explicit and communicated from one to another*.” The RAPM test comprises 48 patterns (questions) divided into two sets (I and II). Set-I contains 12 practice patterns and Set-II contains 36 patterns used to assess cognitive ability. As shown in Figure 1, each pattern contains a 3 × 3-cell structure where each cell contains a geometrical shape, except for the empty right-bottom cell. Eight multiple options are presented as solutions for the empty cell. A score of “1” is assigned for each correct answer and a score of “0” for an incorrect answer. The processing time is 10 and 40 min for sets I and II, respectively.

### Description of dataset I

Procedures adopted for the recording and preprocessing of EEG data are found in our previous studies [7, 8]. After preprocessing, we divided the dataset into two classes as follows: Class 1 included EEG data from eight experimental subjects recorded during their RAPM task performances; Class 2 included EEG data recorded with eyes-open (EO) from the same eight subjects'. As mentioned in the RAPM task description, each subject completed 36 patterns within a specified period. Hence, these EEG recordings were time-marked for the onset of RAPM pattern display, and again at the end of a subject's response, specifically when pressing a button indicating a solution. Each subject's EEG recording was segmented according to the number of patterns solved. Each EEG segment was also considered an observation; thus, producing 36 observations per subject. However, a few patterns went unanswered (unsolved) by some subjects, which EEG segments were excluded from the analysis. We observed 280 observations for Class 1. To maintain a balance between classes, EO and EC (eyes-closed) data for each subject were segmented according to the number of attempted RAPM patterns. As a result, EO data also included 280 observations for Class 2.

### Description of dataset II

EEG dataset utilized in this study was originally reported by Keirn and Aunon (1990) and publically available for reuse. The EEG data was recorded by placing electrodes over C3, C4, P3, P4, O1, and O2 positions according to 10–20 montage and referenced to linked mastoids, A1 and A2. The impedances of all the electrodes were kept below 5 kΩ. The data were digitized at 250 Hz with a Lab Master 12-bit A/D converter mounted on a computer. Seven participants, 21–48 years old, recorded EEG data during cognitive tasks. The data was recorded for duration of 10 s in each trial and each task was repeated five times (for more detail of dataset, see Keirn and Aunon, 1990 original work). For this study, we tested our proposed approach on two cognitive tasks employed from Keirn and Aunon's dataset and described below.

#### Mental multiplication task

The participants were given nontrivial multiplication problems, such as, multiply two multi-digit numbers, and were asked to solve them without vocalizing or making any other physical movements.

#### Mental letter composition task

In this task, the participants were asked to mentally compose a letter to a friend or relative without vocalizing.

### The discrete wavelet transform (DWT)

**Figure 2:**
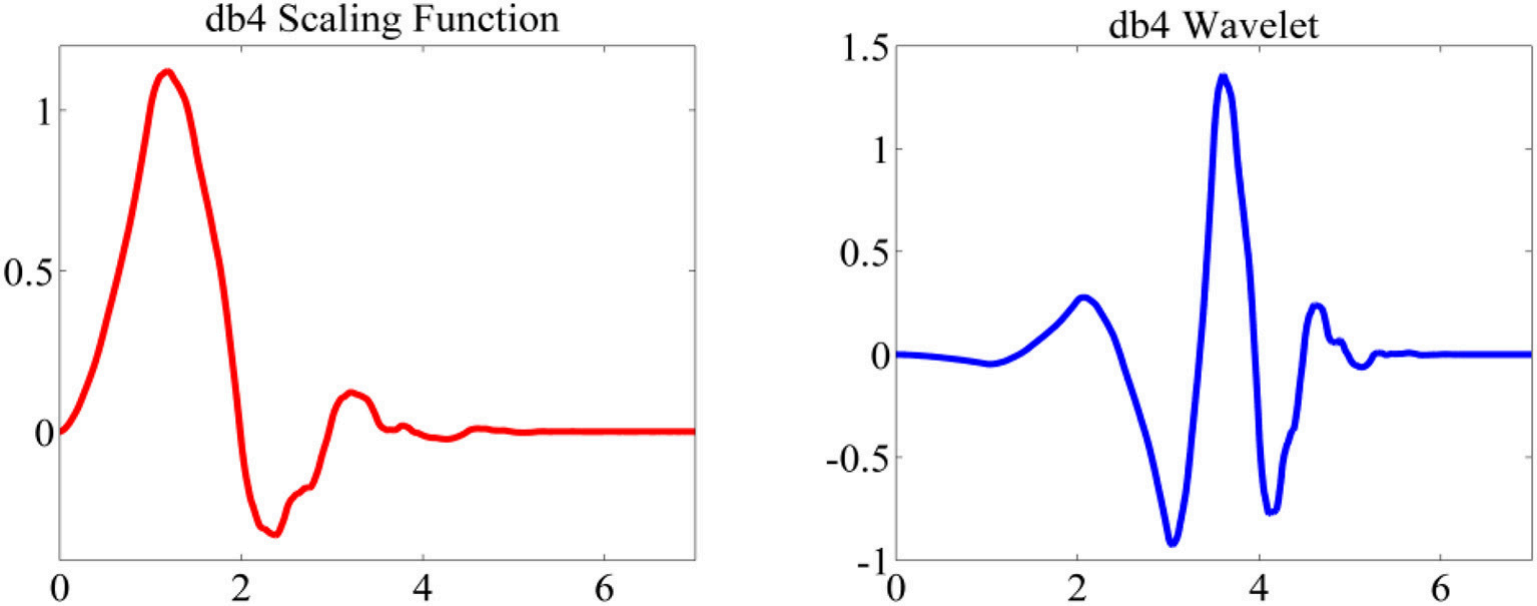
Mother wavelet and scaling function (db4).

Discrete Wavelet Transform decomposition includes successive high and low pass filtering of a time series with a down-sampling rate of 2. The high pass filter [*g*(*n*)] is the discrete “mother” wavelet and the low pass filter [*h*(*n*)] is its mirror version (Subasi, 2007). The “mother” wavelet [Daubechies wavelet (db4)] and corresponding scaling function are shown in Figure 2.

**Figure 3:**
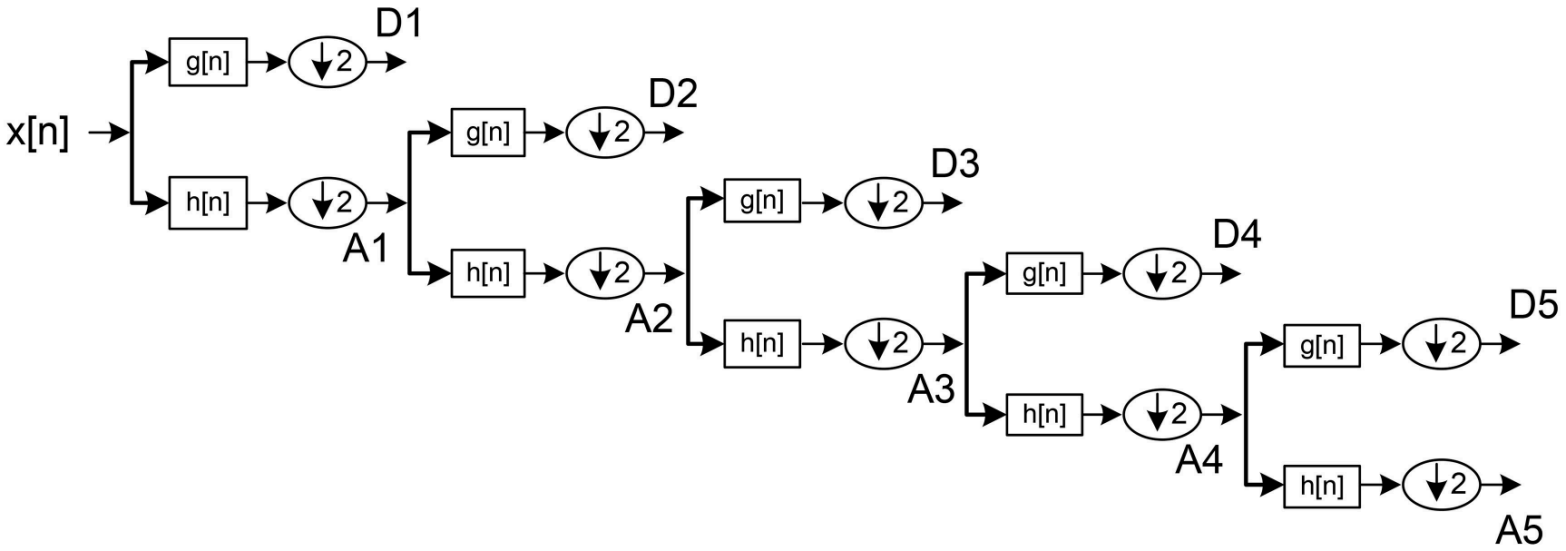
DWT sub-band decomposition.

Outputs from initial high pass and low pass filters are called “approximations” and “detailed” coefficients (A1 and D1), respectively. A1 is disintegrated further and the procedure repeated until reaching a specified number of decomposition levels (see Figure 3; Jahankhani et al., 2006; Subasi, 2007).

The scaling [ φ_j, k_(n)] and wavelet functions [ ψ_j, k_(n)] both depend on low pass and high pass filters, respectively. These are denoted as follows:

(1) $$\phi_{\text{j},\text{k}}\left(\text{n}\right)\;=\;2^{-\text{j}/2}\text{h}\left(2^{-\text{j}}\text{n}-\text{k}\right)$$

(2) $$\psi_{\text{j},\text{k}}\left(\text{n}\right)\;=\;2^{-\text{j}/2}\text{g}\left(2^{-\text{j}}\text{n}-\text{k}\right)$$

Where n = 0, 1, 2, …, *M* − 1; j = 0, 1, 2, …, *J* − 1; k = 0, 1, 2, …, 2^j^ − 1; J = 5; *and*

*M is the length of the signal* (Gonzalez and Woods, 2002).

Approximation coefficients (*A*_*i*_) and detailed coefficients (*D*_*i*_) at the *ith* level are determined as follows (Orhan et al., 2011):

(3) $${\text{A}}_{\text{i}}\;=\frac{1}{\;\sqrt{\text{M}}}\sum_{\text{n}}\text{x}\left(\text{n}\right).\phi_{\text{j},\text{k}}\left(\text{n}\right)$$

(4) $${\text{D}}_{\text{i}}\;=\;\frac{1}{\sqrt{\text{M}}}\sum_{\text{n}}\text{x}\left(\text{n}\right).\psi_{\text{j},\text{k}}\left(\text{n}\right)$$

### Relative and total wavelet sub-band energy

Wavelet energy for each decomposition level (*i* = 1, …, *l*) is determined as follows:

(5) $${\text{E}}_{{\text{D}}_{\text{i}}}=\sum_{\text{j}=1}^{\text{N}}{\left|{\text{D}}_{\text{ij}}\right|}^{2},\text{ i }=\;1,\;2,\;3,\ldots ,l$$

Where *l* = 5, reflects the level of decompositions

(6) $${\text{E}}_{{\text{A}}_{\text{i}}}=\sum_{\text{j}=1}^{\text{N}}{\left|{\text{A}}_{\text{ij}}\right|}^{2},\text{ i }=\;l$$

Therefore, from Equations (5) and (6), total energy can be defined as:

(7) $${\text{E}}_{\text{Total}}=\left(\sum_{\text{i}=1}^{l}{\text{E}}_{{\text{D}}_{\text{i}}}+{\text{E}}_{{\text{A}}_{l}}\right)$$

**Figure 4:**
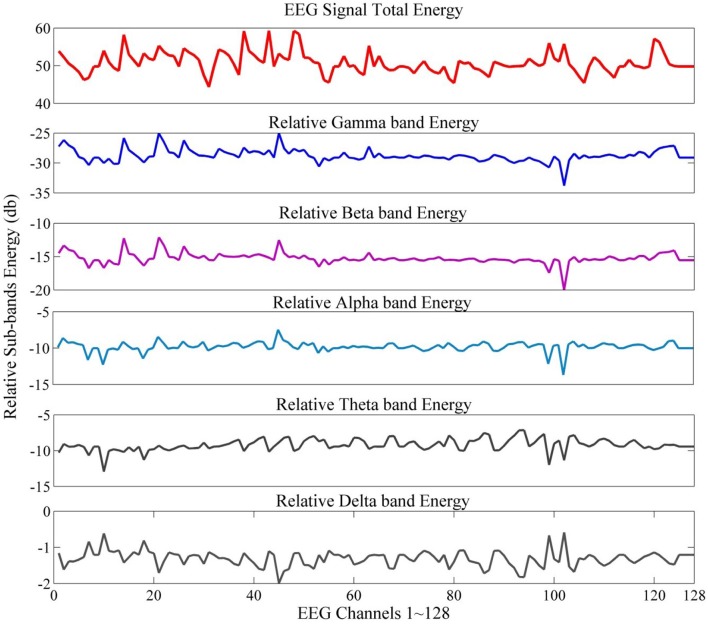
EEG signal energy and relative sub-band energy.

The normalized energy values represent relative wavelet energy (see Figure 4 for an example of total and relative sub-band energy).

(8) $${\text{E}}_{\text{r}}\;=\;\frac{{\text{E}}_{\text{j}}}{{\text{E}}_{\text{Total}}},$$

Where *E*_*j*_ = *E*_*D*_*i* = 1, …, 5__ *or E*_*A*_*i* = 5__.

### Classification algorithms

Machine learning classifiers used in this study are now briefly described.

A classifier utilizes values for independent variables (features) as input to predict the corresponding class to which an independent variable belongs (Pereira et al., 2009). A classifier has a number of parameters that require training from a training dataset. A trained classifier will model the association between classes and corresponding features and is capable of identifying new instances in an unseen testing dataset. We employed the following classification methods to demonstrate the effectiveness of this study's proposed technique.

#### Support vector machine (SVM)

The SVM is a supervised learning algorithm that uses a kernel trick to transform input data into higher dimensional space, after which it segregates the data via a hyper-plan with maximal margins. Due to its ability to manage large datasets, the algorithm is widely used for binary classification problems in machine learning. For more details on SVM, see (Hsu et al., 2003).

#### Multilayer perceptron (MLP)

MLP is a non-linear neural network based method comprising three sequential layers: input, hidden and output, respectively, where the hidden layer transmits input data to the output layer. However, the MLP model can cause over-fitting due to insufficient or excessive numbers of neurons. For our purposes, we employed the MLP model with five *hidden* neurons.

#### Naïve bayes (NB)

The NB classifier provides simple and efficient probabilistic classification based on Bayes' theorem, which posits that extracted features are not dependent. The NB model uses (i) a maximum probability algorithm to determine the class of earlier probabilities, and (ii) a feature's probability distribution from a training dataset. Results are then employed with a maximized posteriori decision tree to find the specific class label for a new test instance (Han et al., 2011).

#### k-Nearest neighbor (k-NN)

The k-nearest neighbor is a supervised learning algorithm that identifies a testing sample's class according the majority class of k-nearest training samples; i.e., a class label is allocated to a new instance of the most common class amongst KNN in the “feature” space. In this study, k value was set to three. See (Pereira et al., 2009; Han et al., 2011) for details on k-NN, SVM, MLP, and NB machine learning classifiers.

#### K-fold cross validation

All classification models in the present work were trained and tested with EEG data and then confirmed using k-fold cross validation, which is a commonly used technique that compares (i) performances of two classification algorithms, or (ii) evaluates the performance of a single classifier on a given dataset (Wong, 2015). It has the advantage of using all instances in a dataset for either training or testing, where each instance is employed for validation exactly once. For our purposes, we used 10-fold cross-validation to train and test extracted features for all classifiers.

### The proposed scheme

**Figure 5:**
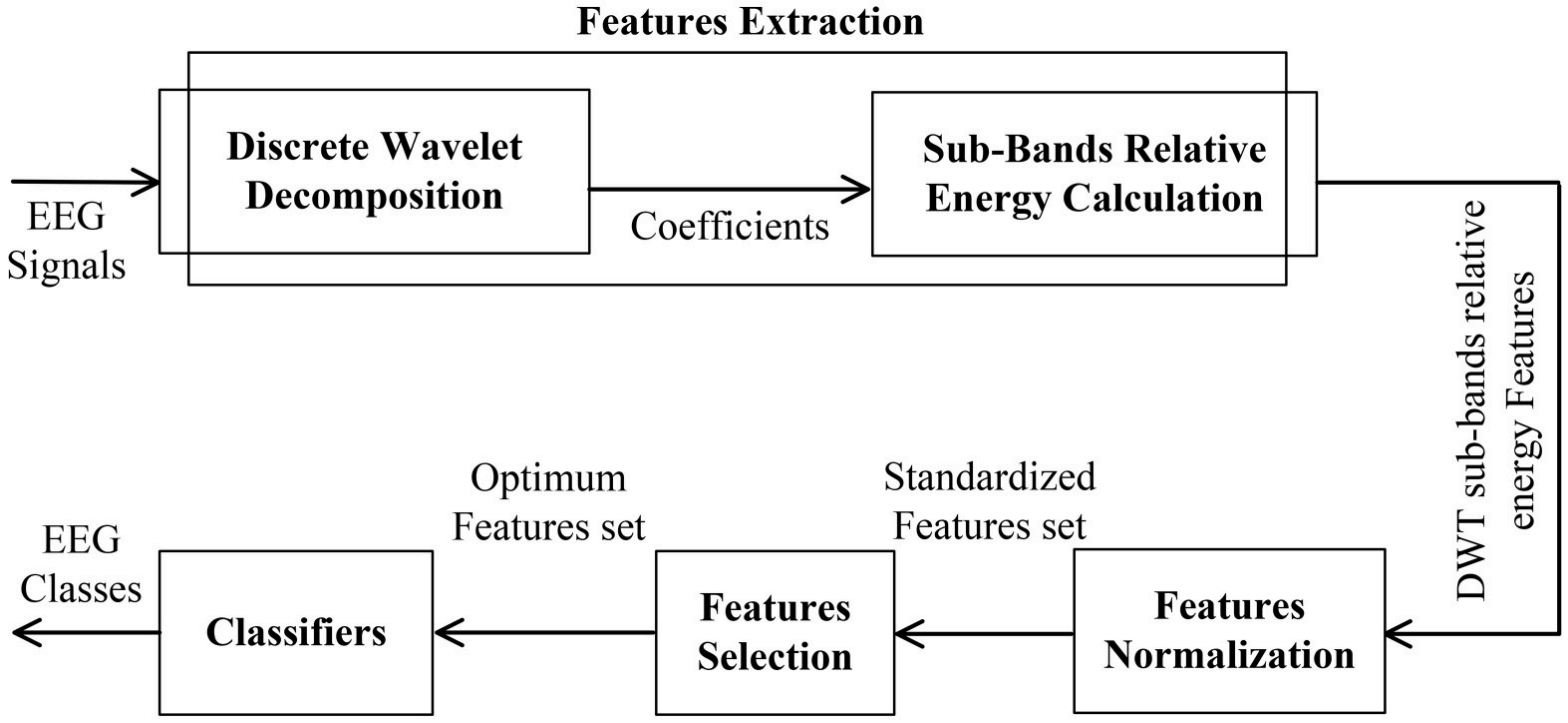
Proposed scheme for feature extraction and classification of EEG signals.

Figure 5 summarizes the proposed feature extraction scheme, which comprises the following steps:

Steps:

a. Feature Extraction
  1. Decomposition of EEG signal into sub-bands using DWT
  2. Computation of each sub-band's relative energy
    Repeat steps 1 and 2 for all channels for each subject and for each segment (question)

b. Feature Visualization and Standardization
  1. Standardization of extracted features to zero mean and unit variance

c. Feature Selection
  1. Application of FDR to the “features” set followed by sorting features in descending order according to the power of discrimination
  2. Selection of subset features above the FDR median of sorted features as listed in step 1
  3. Transforming selected subset features to principal components followed by sorting in descending order according to principal value
  4. Selection of principal components up to 95% variance
  5. Reconstruction of corresponding feature vectors
    Repeat steps 1 to 5 for all sub-bands in the “features” set

d. Feature Classification
  1. Classification of selected feature sets via KNN, SVM, MLP and NB
  2. Evaluation of classifier performances with 10-cross validation and assessing degrees of accuracy, sensitivity and specificity
    Repeat steps 1 and 2 for each sub-band of the “features” set

#### Feature extraction

**Figure 6:**
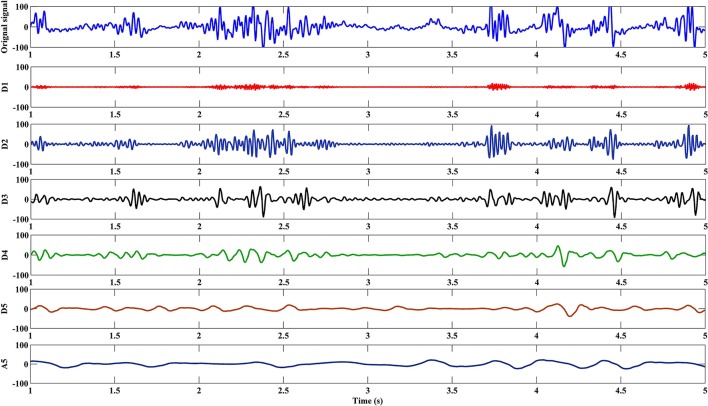
A5 and D1–D5 components of an experimental subject's EEG signal during a cognitive task.

The EEG signal was decomposed into sub-band frequencies by using the discrete DWT with Daubechies 4 Wavelet to level 5. Approximate and detailed coefficients were then computed (see Figure 6 for example). Table 1 presents one channel's sub-band relative energy percentage and frequency range for a single experimental subject. Total and relative sub-band energies were then computed from extracted wavelet coefficients. We calculated relative wavelet energy(*E*_*rD*1_, *E*_*rD*2_, …, *E*_*rA*5_) by using Equation (8).

**Table 1:** Frontal lobe F3 channels.

**Levels**	**Wavelet energy %**	**Wavelet coefficients**	**Frequency bands (Hz)**
1	0.66	D1	62.50–125
2	2.31	D2	31.25–62.50
3	7.63	D3	15.62–31.25
4	10.77	D4	7.81–15.62
5	21.55	D5	3.90–7.81
5	57.12	A5	0–3.90

*Relative energy sub-band percentages and frequency range*.

We computed relative energy features for all experimental subjects and for all data collected from all channels. Accordingly, for dataset I, the feature matrix describing relative energy for a single subject in each EEG condition and for each sub-band (detailed or approximated) becomes:

$$\text{Relative Energy Feature Matrix }(\vec{{\text{F}}_{\text{r}}})\;=\;[{\text{E}}_{\text{rA5}(280\times 128)}]$$

Where, the number of channels is 128; the number of instances in each class is 280; *E*_*rA*5_ represents relative energy at approximation coefficients A5 (0–3.90 Hz). Similarly, feature matrices for D_2_–D_5_ coefficients were identically represented for each class and each experimental subject.

#### Feature visualization and normalization

**Figure 7:**
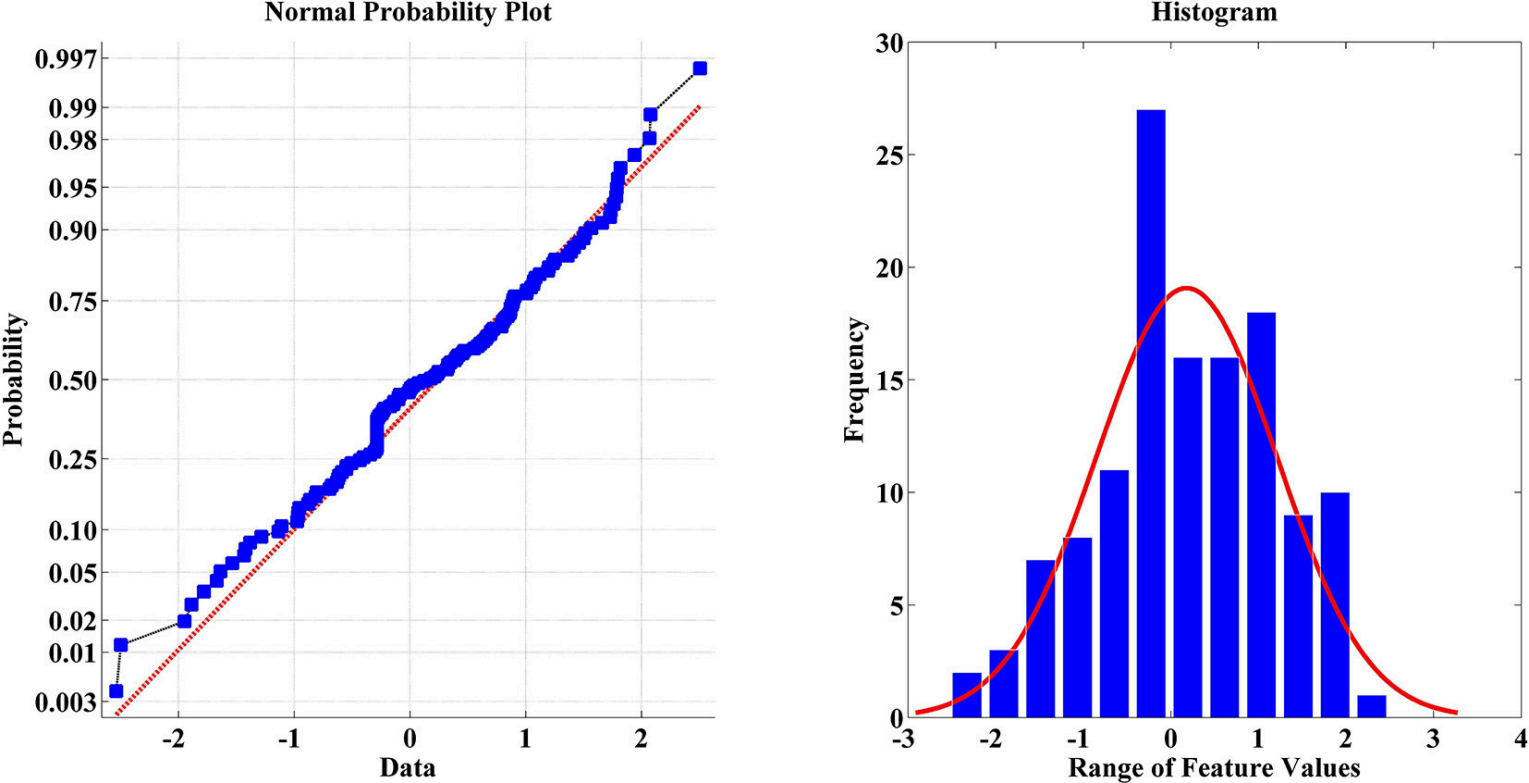
Feature visualization of one sample within a dataset using normal probability and histogram.

Feature visualization is an important step before the application of any normalization method. Features should be visualized to check the distribution of feature values (Figure 7). In this study, features were standardized as follows:

(9) $$\overset{´}{\text{x}}=\frac{\text{x}-\mu }{\sigma }$$

Where *x* is the original feature value; μ is the mean; and σ is the standard deviation of the “features” set, respectively, and x́ is the normalized feature value.

#### Feature selection

The main objective of the feature selection step in pattern recognition is to select a subset from large numbers of available features that more robustly discriminate for purposes of classification (Theodoridis et al., 2010). In this study, FDR and PCA were used to optimize features selection.

FDR is an independent type of class distribution and a quantifier of the discriminating power of individual features between classes. If *m*_1_ and *m*_2_ are mean values, and $\sigma_{1}^{2}$ and $\sigma_{2}^{2}$ are respective variances for a feature in both classes, FDR is defined as:

(10) $$\text{FDR}=\frac{\left({\text{m}}_{1}-{\text{m}}_{2}\right)}{\left(\sigma_{1}^{2}-\sigma_{2}^{2}\right)}$$

PCA (Abdi and Williams, 2010) selects for mutually uncorrelated features; hence, it avoids redundancy in the feature set. In a feature set (*X*), containing (*l*) features of (*N*) examples each, such that (*x*_*i*_ ϵ *R*^*l*^*, i* = 1, 2,…, *N*), PCA aims to linearly transform the feature set and thus obtain a new set of samples (*y*), in which components of (*y*) are uncorrelated per Equation (12).

(11) $$y=A^{T}x$$

Where (*A*) is a transformation matrix or arrangement of singular vectors that correspond to significant singular values.

The PCA process requires a number of steps:

Step 1: Covariance matrix (*S*) of feature matrix (*X*) is estimated:

(12) $$S=\sum_{i=1}^{N}x_{i}x_{i}^{T}$$

Step 2: Singular value decomposition (SVD) is applied to (*S*) as well as to (*l*) singular values and singular vectors (λ_*i*_) and (*a*_*i*_ϵ*R*^*l*^); then (*i* = 1, 2,…, *l*) are computed. Singular values are organized in descending order (λ_1_ ≥ λ_2_ ≥ ··· ≥ λ_l_), after which correct pairing of singular values with corresponding singular vectors are ensured. In addition, (*m*) largest singular values are selected. Normally, (*m*) is selected to include a certain percentage of total energy (95%). Singular values (λ_1_ ≥ λ_2_ ≥ ··· ≥ λ_*m*_) are called (*m*) principal components. Respective (column) singular vectors (*a*_*i*_, *i* = 1, 2,…,*m*) are then used to construct the transformation matrix:

(13) $$\text{A}=[{\text{a}}_{1\;}\;{\text{a}}_{2\;}{\text{a}}_{3\;}\ldots \;{\text{a}}_{\text{m }}]$$

Each *l*-dimensional vector in the original space (*X*), is transformed to an *m*-dimensional vector (*y*), via transformation (*y* = A^T^x). In other words, the ith element of (*y*), i.e. [*y(i)*], is the projection of (*x*) on [*a*_*i*_ ($\text{y}\left(\text{i}\right)={\text{A}}_{\text{i}}^{\text{T}}\text{x}$)].

#### Feature classification

**Figure 8:**
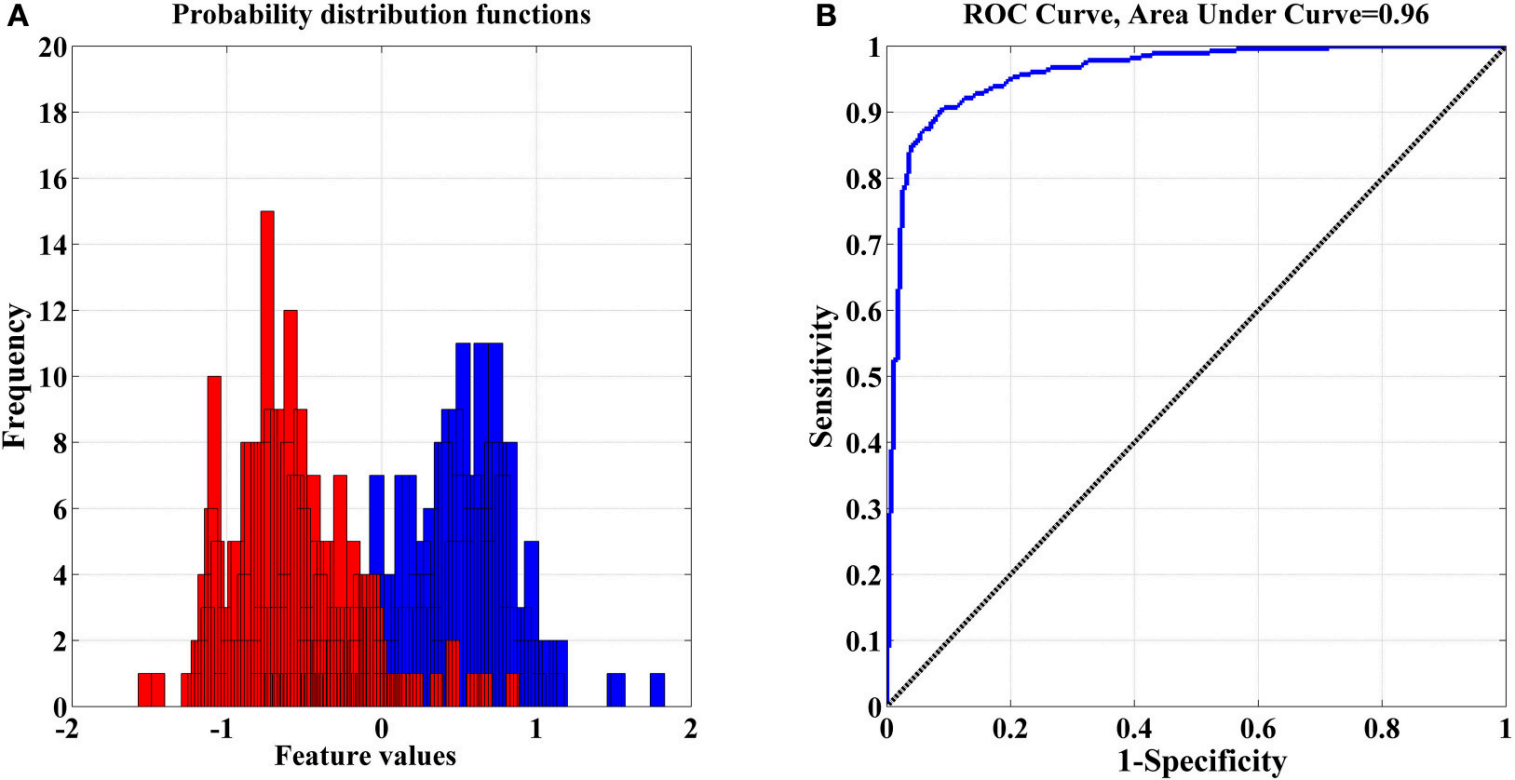
**(A)** Distributions with partial overlap between pdfs of both classes—(eyes open and RAPM); **(B)** Corresponding ROC Curve where 0 denotes complete overlap and 1 indicates complete separation.

The optimized “features” set was visualized by using probability distribution functions (PDFs) and the ROC curve to check for any overlapping in each selected feature for both classes (see Figure 8: EO vs. RAPM). Each selected feature yields a partial overlap and ROC values that are >0.9 or close to 1.0, which confirm the discriminating power of the selected “features” set. Finally, the classifiers, KNN, SVM, MLP, and NB, were employed for the discrimination of both classes.

## Experimental results and discussion

### Experimental setup

According to the 10-fold cross validation process, we divided the dataset into 10 subsets of equal instances with nine subsets employed for classifier training and one subset for classifier testing. The process was repeated 10 times so that each subset was tested for classification. Classifier performance was measured for accuracy, sensitivity, specificity, precision, and the Kappa statistic (Amin et al., 2015a), each defined as follows:

(14) $$\text{Accuracy}=\frac{\text{ Total no}.\text{ of correctly classified instances}}{\text{Total numbers of instances}}\;\times \;100$$

(15) $$\text{Sensitivity}=\;\frac{\text{True Positive}}{\text{True Positive }+\text{ False Negative}}\;\times \;100$$

(16) $$\text{Specificity}=\;\frac{\text{True Negative}}{\text{True Negative }+\text{False Positive}}\;\times \;100$$

(17) $$\text{Precision}=\;\frac{\text{True Positve}}{\text{True Positive + False Positive}}\;\times \;100$$

(18) $$\text{Kappa }(\text{k})\;=\;\frac{\left({\text{P}}_{\text{o}}-{\text{P}}_{\text{e}}^{\text{C}}\right)}{\left(1-{\text{P}}_{\text{e}}^{\text{C}}\right)}$$

Where P_o_ represents the probability of overall agreement between label assignments, classifier and true process; and ${\text{P}}_{\text{e}}^{\text{C}}$ denotes the chance agreement for all labels; i.e., the sum of the proportion of instances assigned to class multiplies in proportion to true labels of that specific class in the data set.

### Classification results

We used DWT to extract relative wavelet energy features for D1–D5 and A5 for both EEG datasets, where dataset I contained two conditions (EO and RAPM) and dataset II contained two cognitive tasks, i.e., mental multiplication task and mental letter composing task. The extracted features were reduced to optimum number of features by using FDR and PCA. We further applied machine learning algorithms, i.e., SVM with RBF kernel, MLP with five hidden layers, KNN with *k* = 3, and NB to classify the extracted features to all decomposition levels (D2–D5 and A5). The detail coefficient D1 reflects high frequency (62.5–125 Hz) components, thus considered as noise and excluded from classification.

#### Results of dataset I

The performance of SVM and KNN classifiers showed 99.11 and 98.21% accuracies each for A5 approximation coefficients, and 98.57 and 98.39% accuracies for D5 details coefficients, respectively, as shown in Tables 2, 3. This impressive performance at level 5 reflects low frequency (0–3.90 Hz) and above low frequency (3.90–7.81 Hz) cognitive domination tasks. MLP and NB classifier accuracies were 97.14 and 89.63%, respectively, for approximated coefficients and 91.60 and 81.07% for detailed at level 5. Results for other performance parameters, i.e., sensitivity, specificity, precision, and the Kappa statistic, were also impressive. Subject-wise classification accuracies for complex cognitive task (RAPM) vs. eyes open (EO) baseline are presented in Tables 4, 5. The mean and standard deviation of accuracies with KNN, SVM, MLP, and Naïve classifiers for approximation coefficients (0–3.90 Hz) are 96.25 ± 2.47, 97.14 ± 1.60, 97.14 ± 1.43, and 96.07 ± 1.19; and detailed coefficients (3.90–7.81 Hz) are 94.10 ± 2.69, 95.35 ± 2.62, 97.32 ± 1.42, and 93.92 ± 2.83, respectively.

**Table 2:** Approximation coefficients (0 – 3.90 Hz) for cognitive tasks.

**Classifier**	**Accuracy (%)**	**Sensitivity (%)**	**Specificity (%)**	**AUC**	**Precision (%)**	**Kappa Statistic**
KNN, *k* = 3	98.21	96.88	99.63	0.98	99.64	0.96
SVM, RBF	99.11	99.28	98.93	0.99	98.93	0.97
MLP, *N* = 5	97.14	96.48	97.83	0.98	97.86	0.94
Naïve	89.63	88.24	90.77	0.94	91.07	0.78

*Relative wavelet energy classification results for level 5*.

**Table 3:** Detailed coefficients (3.90 – 7.81 Hz) for cognitive tasks.

**Classifier**	**Accuracy (%)**	**Sensitivity (%)**	**Specificity (%)**	**AUC**	**Precision (%)**	**Kappa Statistic**
KNN, *k* = 3	98.39	98.22	98.56	0.98	98.56	0.96
SVM, RBF	98.57	97.89	99.28	0.98	99.28	0.96
MLP, *N* = 5	91.60	88.70	94.98	0.92	95.36	0.89
Naïve	81.07	82.71	79.59	0.82	78.57	0.79

*Relative wavelet energy classification results for level 5*.

**Table 4:** Approximation coefficients (0 – 3.90 Hz) for subject wise classification accuracy.

	**KNN**	**SVM**	**MLP**	**BN**
Subject 1	97.14	100	97.14	97.14
Subject 2	98.57	98.57	98.57	95.71
Subject 3	94.28	97.14	94.28	95.71
Subject 4	92.85	95.71	95.71	94.28
Subject 5	92.85	97.14	98.57	95.71
Subject 6	100	98.57	97.14	95.71
Subject 7	97.14	95.71	97.14	95.71
Subject 8	97.14	100	98.57	98.57
Mean	96.25	97.86	97.14	96.07
STD	2.47	1.60	1.43	1.19

*Relative wavelet energy classification results for level 5*.

**Table 5:** Detailed coefficients (3.90 – 7.81 Hz) for subject wise classification accuracy.

	**KNN**	**SVM**	**MLP**	**BN**
Subject 1	90.00	91.42	97.14	91.42
Subject 2	95.71	97.14	95.71	98.57
Subject 3	94.28	95.71	95.71	95.71
Subject 4	90.00	98.57	98.57	92.85
Subject 5	94.28	95.71	97.14	91.42
Subject 6	95.71	91.42	100	91.42
Subject 7	95.71	95.71	97.14	92.85
Subject 8	97.14	97.14	97.14	97.14
Mean	94.10	95.35	97.32	93.92
STD	2.69	2.62	1.42	2.83

*Relative wavelet energy classification results for level 5*.

#### Results of dataset II

For classification of two cognitive tasks, i.e., mental multiplication task and mental letter composing task, MLP and SVM classifiers achieved 89.17% and 86.67% accuracies each for A5 approximation coefficients, and KNN and MLP classifiers achieved 93.33 and 92.50% accuracies for D5 details coefficients, respectively, as shown in Tables 6, 7. These results indicate that the proposed approach is strong enough to perform on public dataset for classification of cognitive tasks. Comparison of the proposed approach with previous work on the same dataset is presented in Table 8.

**Table 6:** Approximation coefficients (0 – 3.90 Hz) for cognitive tasks (mental multiplication vs. mental letter composing).

**Classifier**	**Accuracy (%)**	**Sensitivity (%)**	**Specificity (%)**	**AUC**	**Precision (%)**	**Kappa statistic**
KNN, *k* = 3	82.50	80.95	84.21	0.85	85	0.84
SVM, RBF	86.67	87.93	85.48	0.89	85	0.87
MLP, *N* = 5	89.17	89.83	88.52	0.90	88.33	0.89
Naïve	78.33	77.42	79.31	0.79	80	0.77

*Relative wavelet energy classification results for level 5*.

**Table 7:** Detailed coefficients (3.90 – 7.81 Hz) for cognitive tasks (mental multiplication vs. mental letter composing).

**Classifier**	**Accuracy (%)**	**Sensitivity (%)**	**Specificity (%)**	**AUC**	**Precision (%)**	**Kappa statistic**
KNN, *k* = 3	93.33	91.94	94.83	0.92	95	0.93
SVM, RBF	90	87.50	92.86	0.92	93.33	0.90
MLP, *N* = 5	92.50	90.48	94.74	0.94	95	0.91
Naïve	84.17	82.54	85.96	0.85	96.67	0.83

*Relative wavelet energy classification results for level 5*.

**Table 8:** Comparison of the proposed approach with previous work using EEG dataset recorded by Keirn and Aunon (1990).

**Author**	**Methods**	**Accuracy**
Keirn and Aunon, 1990	Spectral density estimated and classified with Bayes quadratic classifier	81.5
Liang et al., 2006	Feature extracted with autoregressive coefficients with SVM classifier. Here average classification accuracy is reported using 1-against-1 SVM for multiplication task	54.77
Zhang et al., 2010	EEG Power estimated with Fourier transform and classified using Fisher discriminant analysis	72.4
Vidaurre et al., 2013	Feature extracted using autoregressive and classified with SVM classifier	73
Hariharan et al., 2014	Feature extracted using Stockwell transform and classified with KNN	84
Hendel et al., 2016	Signal energy was estimated with DWT and features were classified with SVM classifier. Here reported averaged classification accuracy for multiplication task	84.73
Dutta et al., 2018	Feature extracted with combination of multivariate empirical mode decomposition (MEMD) and phase space reconstruction and classified using LS-SVM with RBF Kernel	83.33
This study	DWT used with computed relative sub-band energy features; features were standardized; Fisher's discriminant ratio; principal component analysis were adopted for optimized feature selections; SVM, MLP, KNN and Naïve Bayes classifiers used for classification	93.33

*Here, the results were compared for two class problem (mental multiplication vs. mental letter composing)*.

## Discussion

This study used a “pattern recognition” based approach to classify EEG signals that were recorded during resting and active cognitive states of consciousness. Using SVM, MLP, KNN, and NB classifiers for both conditions, we classified extracted relative wavelet energy features for D2–D5 and A5. Classification results were not prominently evident for all decomposition levels. Results for the relative energy of approximation and detailed coefficients at level 5 showed the highest performances for cognitive task dominations at low frequency (0–3.90 Hz) and above low frequency (3.90–7.81 Hz; Tables 2, 3, 7, 8).

These results indicate that relative wavelet energy for low frequency (0–3.90 Hz) and above low frequency bands (3.90–7.81 Hz) is a useful feature for EEG classification of cognitive tasks as well as separation of baseline (eyes open) and complex cognitive task. These results confirm DWT's ability to compactly represent EEG signals and compute total and relative energy levels for different frequency bands. The normalization process reduced the non-Gaussianity of extracted features. Furthermore, the use of the proposed feature selection approach minimized non-significant features from a “features” set before feeding it to classifiers, which reduces computational cost.

Results of Dataset I are not exactly comparable with previous work except the authors' own work published in 2015 in which the same EEG data was used as described in dataset I. However, dataset II is a publicly available dataset, which is used to compare with previous work done on the same dataset. The notion reflected by the studies is the discriminatory performance levels (accuracy) achieved by various quantitative analytical approaches to classify two different cognitive activities using EEG signals, for example mental multiplication and mental letter composing(Liang et al., 2006; Zhang et al., 2010; Vidaurre et al., 2013; Dutta et al., 2018). These studies employed the EEG data originally reported by Keirn and Aunon (1990) and applied different feature extraction and classification algorithms. Here, the authors reported the results of previous studies for comparison of only two cognitive tasks, i.e., mental multiplication and mental letter composing, for example 81.5% classification accuracy reported by Keirn and Aunon (1990) for classification of mental multiplication from mental letter composing (see Table 8 for a detailed summary of these methods and results as reported in the literature). The cited authors reported low classification accuracy rates and used complex classification models such as, neural network or kernel-based classifiers. Our proposed scheme, as demonstrated in the present study, yielded higher accuracy rates with a SVM and KNN, indicating superior discriminatory performances when assessing mental tasks. In addition, the present study achieved high classification accuracy than our previous study using the same EEG dataset. Therefore, the proposed method employed feature normalization and optimization modalities that appear to have yielded a more efficient and reliable solution.

## Limitations

There are some limitations in the present study, which should be highlighted for future research. The authors used dataset from their previous study and a small public dataset. However, a large public dataset can validate the robustness of the proposed method for EEG signals classification. In addition, EEG recorded during a cognitive task is relatively easy to separate from a baseline EEG than EEG signals recorded in two different cognitive tasks. In future study, we will extend the application of the proposed method to clinical datasets, such as, classification of ictal vs. inter-ictal or normal EEG patterns.

## Conclusion

The authors presented a pattern recognition based approach for classification of cognitive tasks using EEG signals. The DWT was applied to EEG signals for decomposition. Classification results were superior to our previous study (Amin et al., 2015a) for dataset I as well as previous work done on dataset II. The experimental results validated the proposed scheme. These outcomes suggest promising potential for the method's application to clinical datasets as a beneficial adjunct for discrimination between normal and abnormal EEG patterns, as it is able to cope with variations in non-stationary EEG signals via the localization characteristic of the WT (Rosso et al., 2001). Finally, in this study, the combination of DWT with FDA and PCA techniques provide a robust feature extraction approach for classification of cognitive tasks using EEG signals.

## Ethics statement

This research study was approved by the Research Coordination Committee of Universiti Teknologi PETRONAS, Malaysia. All the participants had signed the informed consent form before starting the experiment. The consent document had a brief description of this research study concerning humans.

## Author contributions

HUA and ASM developed the methodology. WM and ARS performed the analysis and the results interpretation. HUA and NMS drafted the manuscript. All authors read and approved the manuscript.

### Conflict of interest statement

The authors declare that the research was conducted in the absence of any commercial or financial relationships that could be construed as a potential conflict of interest.
